# Supplementary material for: Bioreversible Anionic Cloaking Enables Intracellular Protein Delivery with Ionizable Lipid Nanoparticles
Source: ACS Cent Sci. 2024 May 14;10(6):1179–90. doi: 10.1021/acscentsci.4c00071 (PMC11212127; doi:10.1021/acscentsci.4c00071)
Supplement: Supplementary file 2 — oc4c00071_si_002.pdf [file oc4c00071_si_002.pdf]

oc-2024-00071d.R1

Name: Peer Review Information for "Bioreversible anionic cloaking enables intracellular protein delivery with ionizable lipid nanoparticles"

First Round of Reviewer Comments

Reviewer: 1

Comments to the Author

This manuscript introduces a platform strategy for the reversible functionalisation of proteins to allow their delivery to cells via commercial lipid nanoparticle formulations. The manuscript covers a wide range of proteins, their functionalisation and delivery, including in-vivo studies using mCherry. Overall the manuscript is well written and generally supported by the data presented. There are however some concerns this reviewer has, discussed below.

The major concern this reviewer has with the work relates to the fundamental claim/proposed finding of the work. The authors, throughout the manuscript, state that their system delivers proteins to the cells cytoplasm, where it can then perform its desired function. While this reviewer finds some of the evidence convincing in this regard (notably work with antibodies and RNAse), there are a number of experiments in the early stages of the manuscript and the in-vivo studies, that remain unconvincing. For example, in Figure 2g (and associated discussion) the authors claim the confocal microscopy images show delivery to the cell. Can this image alone be used (without further dyes/images) to prove cell internalisation? While the cells appear fluorescent, can attachment to the cell membrane be ruled out from this image? For example, a cell-surface binding/attachment mechanism (without cell internalisation), could be used to explain much of the other data in Figure 2 and 3 related to cells, and the authors have provided no clear evidence of delivery (internally) to cells to counteract other mechanisms. The authors need to provide counter stains and/or z-stacked confocal images to confirm cell internalisation. This point is more clearly evidenced in Figure 3g where the green fluorescence does not overlap with the blue cell stain. Instead making it appear that the LNPs containing the protein are attached to the cell surface and/or aggregated around the cells, rather than delivering the protein to the cells. Hoescht stained images should be added to Figure 2g and brightfield images added to Figure 3g.

While discussing the fluorescent microscopy images, the authors include only 1 image for each condition. There are no additional images in the ESi of the manuscript. It is therefore impossible for this reviewer to understand if the images shown are representative or merely cherry-picked to

support the claims of the authors. Further images (3+ per condition), including at lower magnifications, should be included in the ESI of the manuscript to support the authors claims.

Some more minor comments on the manuscript:

- The authors use initial studies to "prove" cell delivery and as discussed above this isn't convincing to this reviewer. If cellular delivery is questioned, and the mechanism of cell-membrane binding is proposed, further evidence throughout the manuscript comes into question. For example, in the in-vivo work, the authors fail to include a mCherry-SL4 only control. The authors themselves state that protein binding to a negatively charged LNP in-vivo can change its distribution, it was therefore surprising that this control was not performed as mCherry-SL4 will behave very differently to mCherry (unfunctionalized) and the true comparison would be to the mCherry-SL4. mCherry-SL4 could distribute similarly to the mCherry-SL4/LNPs and as no evidence is provided from this study for cell internalisation, it could be that the mCherry-SL4 also binds in-vivo proteins and is distributed to the lungs etc with longer lifetimes. Can the authors comment on this and/or provide evidence from the in-vivo studies for cell internalisation, for example, histology of the isolated organs and fluorescent imaging?

- The authors use DTT throughout the manuscript to cleave the disulfide bonds of their sulfonate coating compound. A number of protein utilise disulfide bonds, can the authors comment on how such a strategy would affect which proteins could be used using their approach? Would some proteins be ineffective in the cell cytosol due to a similar disulfide reduction mechanism? Discussion would be valuable to the reader in the text.

- The authors state in the introduction of the manuscript that "instability in serum" is a key barrier to such delivery approaches. Yet the authors do not test the serum stability of their formulations. In Figure 6a, serum stability is mentioned but there is no discussion in the text on each protein nor description of how this data was collected in the ESI. This needs to be added to the manuscript.

- Figure 6 (and associated discussion, page 14 line 7-1): It is unclear (and not referenced in the text) why a size <250nm is considered optimal. This is information that should be supported with appropriate references and discussion.

- Can the authors comment on how a RNAse A with uncleavable linkages (Figure S6f), and low activity (Figure S6g), can still lead to cell viability decreases of around 25% (Figure 4c)?

Minor comments to be addressed on the manuscript:

- Page 7, line 34 – Figure S1 doesn't seem to include any carboxylate moieties? But this is claimed in the text.

- Page 7, line 42 and 44 – Figure S4c need to be clearer in the image that this is related to CL4 and a, b of same figure is SL4.

- Supplementary materials – confocal microscopy section: 'Hoescht' is spelt incorrectly.

- Figure 1 appear pixelated and should be of a higher resolution.

- Gels appear also blurred/out of focus. This makes defining the findings of the gels difficult and images should be improved.

- Figure 4 c to e, it is not clear from the figure that the samples discussed in this figure also include MC3 LNPs for delivery. This is confusing to a reader and should be corrected.

- Figure S6 d – x-axis is unlabelled.

- Page 15 line, 14 – 'approaches' is spelt incorrectly

- Figure S8: in-figure labels/captions are unreadable. This needs to be modified/improved.

- In order to allow the reader to understand the included NMRs, proton environments should be labelled and peaks assigned in the NMR images. Some NMRs contain peaks that are not integrated or explained (i.e. Figure S16) leading a reader to question what they are and their relevance/importance.

Reviewer: 2

Comments to the Author

This manuscript introduces a method for protein delivery using MC3 lipid nanoparticles. The authors utilize disulfide bonds to conjugate lysines on the protein surface with negatively charged sulfonated compounds. This modification enables electrostatic interactions with ionizable cationic lipids, facilitating protein encapsulation in MC3 LNPs supplemented with DOTAP. In the cytosol, sulfonated compounds are cleaved, ensuring complete protein functionality as evidenced by the delivery of RNase A and anti-β-Catenin IgG proteins to cancer cell lines, along with in vivo biodistribution studies using mCherry-SL4 protein, supporting the potential of this anionically cloaked protein delivery platform to deliver intracellular therapeutic protein agents. Overall, the work is comprehensive and exciting.

Q1: A high equivalent of sulfonated conjugates may lead to an excessive amount of free compounds. Did you purify the conjugated proteins before formulating the LNPs? In addition, the impact of the sulfonated compound on cell viability should be included.

Q2: What is the reasoning for choosing SL4 over the other two compounds for conjugation? In Figure 2f, there is no indication of a statistical significance between SL2a and SL4 at 30 eq.

Q3: For the in vitro study, DSG-PEG was used (page 6). However, PEG-DMG-2000 was used in the in vivo study (page 14). What's the rationale to use these two different PEG lipids?

Q4: Please include the LNP characterization with protein cargo in terms of size, zeta potential, and encapsulation efficiency.

Q5: The manuscript did not mention Supplementary Fig. 4e-h.

Q6: Please be consistent in adding statistical significance to SI figures.

Reviewer: 3

Comments to the Author

This is an excellent study. I think this should be published as is. This is beautifully done!

Author's Response to Peer Review Comments:

March 22, 2024

To the Editor and Reviewers:

We thank the editors and reviewers for their thoughtful consideration of our manuscript. We understand the time that it takes to give critical and constructive feedback and we are grateful for the detailed review and comments. In the text that follows, we have responded to questions raised by the reviewers in a point-by-point fashion and have made corresponding revisions to our manuscript. Revisions to the manuscript and SI can be seen in the word document with track

changes on. We believe these changes have significantly improved the manuscript (thank you) and that it is now worthy of publication in ACS Central Science.

Response to comments raised by the reviewers.

Reviewer: 1
Recommendation: Publish in ACS Central Science after minor revisions noted.

Comments: This manuscript introduces a platform strategy for the reversible functionalisation of proteins to allow their delivery to cells via commercial lipid nanoparticle formulations. The manuscript covers a wide range of proteins, their functionalisation and delivery, including invivo studies using mCherry. Overall, the manuscript is well written and generally supported by the data presented. There are however some concerns this reviewer has, discussed below.

> Response: We appreciate Reviewer 1's positive assessment of our work and recognition that "*the Manuscript is well written and generally supported by the data presented*." We also appreciate Reviewer 1's recommendations for how to improve the manuscript, which we have done through a combination of new experiments and revisions to the manuscript text. In the section that follows, we have addressed the aspects that Reviewer 1 encouraged us to consider.

Feedback #1: The major concern this reviewer has with the work relates to the fundamental claim/proposed finding of the work. The authors, throughout the manuscript, state that their system delivers proteins to the cells cytoplasm, where it can then perform its desired function. While this reviewer finds some of the evidence convincing in this regard (notably work with antibodies and RNAse), there are a number of experiments in the early stages of the manuscript and the in-vivo studies, that remain unconvincing. For example, in Figure 2g (and associated discussion) the authors claim the confocal microscopy images show delivery to the cell. Can this image alone be used (without further dyes/images) to prove cell internalisation? While the cells appear fluorescent, can attachment to the cell membrane be ruled out from this image? For example, a cell-surface binding/attachment mechanism (without cell internalization), could be used to explain much of the other data in Figure 2 and 3 related to cells, and the authors have provided no clear evidence of delivery (internally) to cells to counteract other mechanisms. The authors need to provide counter stains and/or z-stacked confocal images to confirm cell internalisation. This point is more clearly evidenced in Figure 3g where the green fluorescence does not overlap with the blue cell stain. Instead making it appear that the LNPs containing the protein are attached to the cell surface and/or aggregated around the cells, rather than delivering the protein to the cells. Hoescht stained images should be added to Figure 2g and brightfield images added to Fig. 3g.

> Response: We thank Reviewer 1 for pointing out the need for a clearer demonstration of protein internalization, in particular for the early stages of the work focused on sfGFP. We have added a supplementary Figure (Figure S7) to show brightfield image of the same images shown in Figure 3g as requested. Unfortunately, we did not use Hoechst staining for the image in Figure 2g so we

don't have that image to add. To resolve this issue and provide further support for internalization, we have now revised the supporting information with new confocal images of cells transfected with sfGFP and stained with lysosome and microtubule markers (Figure S5). Brightfield images are included. Diffuse GFP signal indicative of cytosolic localization is seen in the cells treated with MC3 LNPs formulated with sfGFP-SL4, albeit at a lower intensity as compared to the punctate fluorescence likely coming from proteins trapped in endosomes. We would like to point out that these colocalization experiments are consistent with previously reported literature on delivery of siRNAs encapsulated in LNPs in which excellent in vitro gene silencing efficacy was achieved even though a high degree of vesicular localization was observed by microscopy as shown in the figure below from Sahay et al. (Nat Biotechnol, 2013; 31, 653-8):

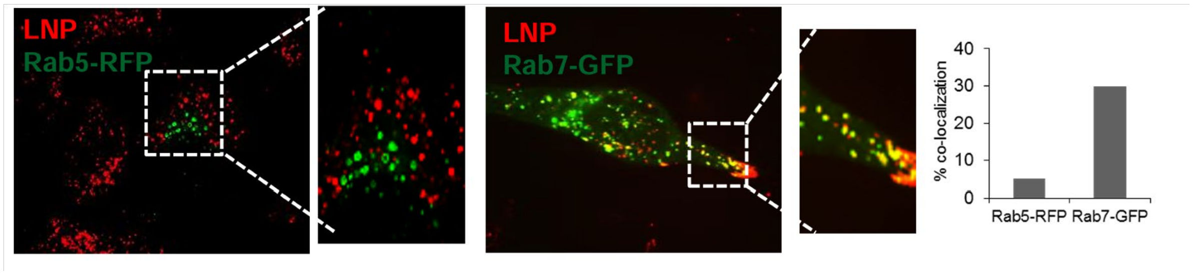

Images from Sahay et al. (Nat Biotechnol, 2013; 31, 653-8)

Additionally, our newly added confocal microscopy images (Figure S5) demonstrate that most of the sfGFP fluorescence is a result of internalized protein and not from any membranebound populations, thus providing confidence that data gathered from uptake experiments are a result of internalized proteins. While it is true that membrane-bound proteins may lead to false positive signals for intracellular protein delivery, we did not observe any "ring"-like signals on the cell periphery that is typically associated with membrane-bound fluorescent probes, such as in the image below from Sorkin *et al.*:

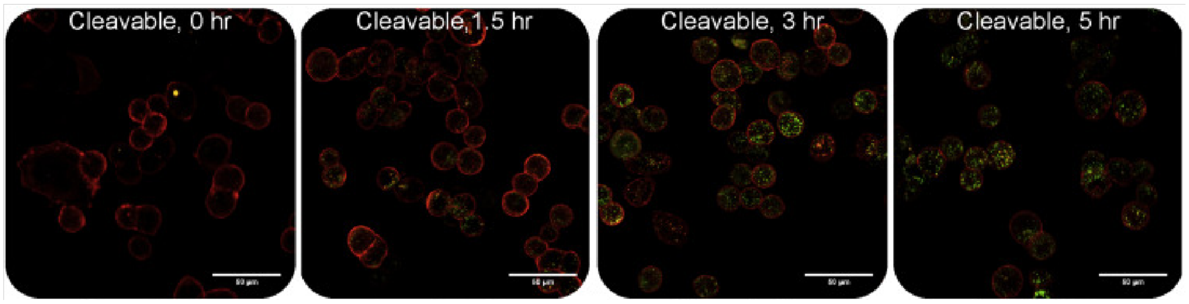

Image from Sorkin et al. (Cell Chem Biol 2019; 26, 1643-51). Red ring (fluorophore tagged antibody) demonstrating surface bound fluorescent signal. Green shows internalized signal.

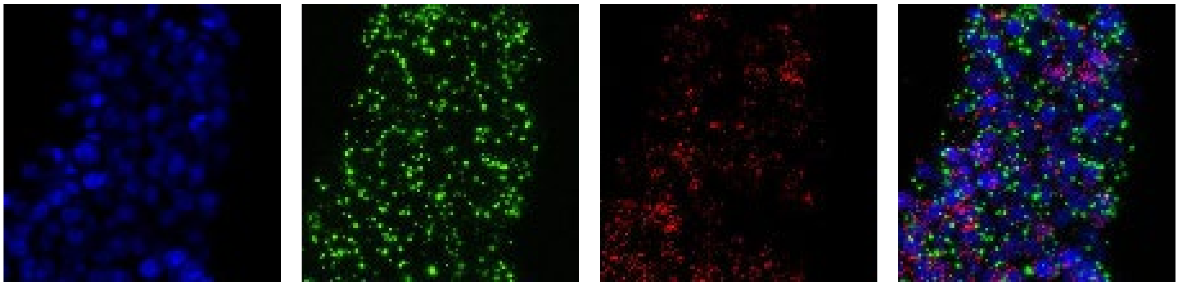

Confocal image from this work demonstrating punctate and partial diffuse intracellular signal.

We note the limitations of the uptake experiments performed with fluorescent proteins with regards to explicitly evaluating cytosolic delivery. However, our ultimate goal was to corroborate cytosolic delivery using functional assays as were performed using RNase A and anti-β-catenin IgG antibodies. We believe these latter experiments provide compelling evidence of protein bioactivity as a direct consequence of cytosolic delivery.

Feedback #2: While discussing the fluorescent microscopy images, the authors include only 1 image for each condition. There are no additional images in the ESi of the manuscript. It is therefore impossible for this reviewer to understand if the images shown are representative or merely cherry-picked to support the claims of the authors. Further images (3+ per condition), including at lower magnifications, should be included in the ESI of the manuscript to support the authors claims.

Response: This is a good point. To address this concern, we have included additional confocal microscopy experiments, including at lower magnifications, in Figure S5 as well as the original uncropped images of Figure 3g in Figure S7 to support our claims.

Feedback #3: The authors use initial studies to "prove" cell delivery and as discussed above this isn't convincing to this reviewer. If cellular delivery is questioned, and the mechanism of cellmembrane binding is proposed, further evidence throughout the manuscript comes into question. For example, in the in-vivo work, the authors fail to include a mCherry-SL4 only control. The authors themselves state that protein binding to a negatively charged LNP in-vivo can change its distribution, it was therefore surprising that this control was not performed as mCherry-SL4 will behave very differently to mCherry (unfunctionalized) and the true comparison would be to the mCherry-SL4. mCherry-SL4 could distribute similarly to the mCherry-SL4/LNPs and as no evidence is provided from this study for cell internalisation, it could be that the mCherry-SL4 also binds in-vivo proteins and is distributed to the lungs etc with longer lifetimes. Can the authors comment on this and/or provide evidence from the in-vivo studies for cell internalisation, for example, histology of the isolated organs and fluorescent imaging?

Response: We thank the reviewer for this comment. However, questioning cellular delivery would require ignoring much of the consequential functional data for RNase A and IgG that proves cytosolic delivery (as their functions require cytosolic presence of the protein).

Furthermore, our response above with new confocal images show that these particles deliver intracellularly and are not primarily membrane bound. Nonetheless, the point the reviewer makes for the in vivo experiment is well taken. For the in vivo biodistribution experiments with mCherry-SL4, we dialyzed the LNP mixtures using a dialysis filter containing a 100-kDa MWCO, which removes unencapsulated mCherry-SL4 as shown in the gel below:

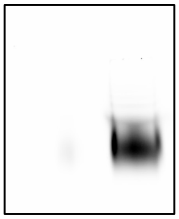

This new native gel shows encapsulation of mCherry-SL4 in MC3 LNPs following dialysis, with and without treatment with Triton-X 100 as indicated. As seen, there is no “free” mCherrySL4 in the formulation after dialysis. Therefore, we do not believe that the LNP samples contain any unencapsulated mCherry-SL4, and we are confident that the signals that we do see from mCherry experiments are solely due to the distribution of the encapsulated mCherrySL4 in LNPs.

We have provided additional immunofluorescence images of histologically sectioned lung tissues in the main text of the manuscript and revised Figure 6e and Figure S10, which demonstrate cellular internalization of LNP-delivered mCherry in vivo. We also previously demonstrated that sfGFP-SL4 was unable to undergo cell internalization in the presence of serum supplemented media (Figure 3a) so we are confident that mCherry-SL4 could not by itself undergo internalization into cells in vivo following protein association. Again, we show in our native gel above that our formulation does not contain free mCherry-SL4.

Feedback #4: The authors use DTT throughout the manuscript to cleave the disulfide bonds of their sulfonate coating compound. A number of protein utilise disulfide bonds, can the authors comment on how such a strategy would affect which proteins could be used using their approach? Would some proteins be ineffective in the cell cytosol due to a similar disulfide reduction mechanism? Discussion would be valuable to the reader in the text.

Response: Reviewer 1 raises a valid point regarding the delivery of disulfide-linked proteins into cells using the anionic-cloaking strategy. Many proteins, including the ones tested in this study (RNase A and IgGs), contain disulfide bonds that might be reduced in the cytoplasmic environment. However, disulfide reduction is a reversible process and may result in transient populations of proteins that retain their disulfide linkages in the cytosol. Additionally, the functional delivery experiments shown for both RNase A and IgGs demonstrate bioactivity following intracellular delivery, suggesting that a significant proportion of the delivered proteins retained biological activity and thus their disulfide linkages must have remained intact. Additional information related to this point has been added to the discussion of the text.

Feedback #5: The authors state in the introduction of the manuscript that "instability in serum" is a key barrier to such delivery approaches. Yet the authors do not test the serum stability of their formulations. In Figure 6a, serum stability is mentioned but there is no discussion in the text on each protein nor description of how this data was collected in the ESI. This needs to be added to the manuscript.

Response: We performed all in vitro experiments in media supplemented with serum, the results of which demonstrate robust delivery efficiencies for all tested proteins. However, for in vivo biodistribution experiments with mCherry, we were especially cautious of the effects of serum on the stability of the LNP formulations and their ability to encapsulate proteins during systemic circulation. Our goal was to corroborate transfection efficiencies from in vitro delivery experiments with the stability of the same LNP-mCherry-SL4 formulations in the presence of mouse serum.

We thank the reviewer for highlighting the absence of methods and data used in calculating serum stability of the LNPs screened for biodistribution studies and have added this information to the Methods section as well as in the Supporting Information.

Feedback #6: Figure 6 (and associated discussion, page 14 line 7-1): It is unclear (and not referenced in the text) why a size <250nm is considered optimal. This is information that should be supported with appropriate references and discussion.

Response: We have added the following discussion with relevant references in the manuscript on particle size: "Generally, clinical LNP formulations of sizes 50-200 nm have been shown to exhibit prolonged circulation half-lives, decreased renal filtration, and efficient endocytic uptake into cells…"

Feedback #7: Can the authors comment on how a RNAse A with uncleavable linkages (Figure S6f), and low activity (Figure S6g), can still lead to cell viability decreases of around 25% (Figure 4c)?

Response: Decreases in cell viability from transfections of RNase A could still arise from partial activity of conjugated RNase A. The reason for this is that the conjugation chemistry is statistical in nature, and thus there while the average degree of labeling is 3-4, there will be some RNAse A proteins with a lower degree of labeling of 1 in the mixture that could have residual activity. This is corroborated by Figure S6g, where partial RNase A activity remains following conjugation with non-cleavable SL4.

Minor comments to be addressed on the manuscript:

1. Page 7, line 34 – Figure S1 doesn't seem to include any carboxylate moieties? But this is claimed in the text.

Response: We thank the reviewer for catching this error. It has been corrected in the supplementary information.

2. Page 7, line 42 and 44 – Figure S4c need to be clearer in the image that this is related to CL4 and a, b of same figure is SL4.

   Response: The images have been replaced with higher quality versions in the supplementary figure.

3. Supplementary materials – confocal microscopy section: 'Hoescht' is spelt incorrectly.

   Response: We thank the reviewer for catching this error. It has been corrected in the supplementary information.

4. Figure 1 appear pixelated and should be of a higher resolution.

5. Gels appear also blurred/out of focus. This makes defining the findings of the gels difficult and images should be improved.

   Response: Low-resolution images were likely a consequence of possible exporting errors in the initial submission. Importantly, the revised manuscript was confirmed to ensure the image quality was retained/restored.

6. Figure 4 c to e, it is not clear from the figure that the samples discussed in this figure also include MC3 LNPs for delivery. This is confusing to a reader and should be corrected.

   Response: Figure captions have been added to clarify which samples were delivered with MC3 LNPs.

7. Figure S6 d – x-axis is unlabelled.

   Response: We thank the reviewer for catching this error. It has been corrected in the supplementary information.

8. Page 15 line, 14 – 'approaches' is spelt incorrectly

   Response: We thank the reviewer for catching this error. It has been corrected in the main text.

9. Figure S8: in-figure labels/captions are unreadable. This needs to be modified/improved.

Response: The figures have been enlarged and upscaled to enhance clarity.

10. In order to allow the reader to understand the included NMRs, proton environments should be labelled and peaks assigned in the NMR images. Some NMRs contain peaks that are not integrated or explained (i.e. Figure S16) leading a reader to question what they are and their relevance/importance.

Response: Proton peaks have been assigned in the included NMR plots. Extraneous/irrelevant peaks are likely from minor impurities in the purified samples.

Reviewer: 2

Recommendation: Publish in ACS Central Science after minor revisions noted.

This manuscript introduces a method for protein delivery using MC3 lipid nanoparticles. The authors utilize disulfide bonds to conjugate lysines on the protein surface with negatively charged sulfonated compounds. This modification enables electrostatic interactions with ionizable cationic lipids, facilitating protein encapsulation in MC3 LNPs supplemented with DOTAP. In the cytosol, sulfonated compounds are cleaved, ensuring complete protein functionality as evidenced by the delivery of RNase A and anti-β-Catenin IgG proteins to cancer cell lines, along with in vivo biodistribution studies using mCherry-SL4 protein, supporting the potential of this anionically cloaked protein delivery platform to deliver intracellular therapeutic protein agents. Overall, the work is comprehensive and exciting.

Response: We are grateful for Reviewer 2's thoughtful review of our work and recognition that "the work is comprehensive and exciting." Below, we have addressed the aspects that Reviewer 2 encouraged us to consider.

Feedback #1: A high equivalent of sulfonated conjugates may lead to an excessive amount of free compounds. Did you purify the conjugated proteins before formulating the LNPs? In addition, the impact of the sulfonated compound on cell viability should be included.

Response: We purified the protein reaction mixtures by dialyzing against 1X PBS for 24 hours using dialysis cups containing a 3.5 kDa MWCO, with 2-3 changes of the 1X PBS during the course of the dialysis. Doing so should remove most if not all of the unreacted compounds from the mixture.

Cell viability following transfections with sfGFP-SL4 and mCherry-SL4 using LNPs remained above 90% in all cases, indicating that both the uptake of the LNPs through cell membranes as well as the cytoplasmic delivery of the sulfonated proteins was not cytotoxic. While very high

intracellular concentrations of sulfonated compounds could have an adverse effect on cell viability, we believe that the nanomolar concentrations tested in this study will have minimal effects on cellular health and metabolism largely due to the fact that the charged compounds will likely be buffered by millimolar concentrations of salts in the cytoplasmic environment.

Feedback #2: What is the reasoning for choosing SL4 over the other two compounds for conjugation? In Figure 2f, there is no indication of a statistical significance between SL2a and SL4 at 30 eq.

Response: We selected SL4 due to the valency of sulfonate groups of the tetrasulfonated compound. While conjugation and delivery were similar for sfGFP using all three compounds, we reason that using SL4 for subsequent applications will allow us to install higher degree of anionic sulfonates per lysine attachment, which may be beneficial for site-specific attachments on proteins and peptides. Furthermore, data in Figure 2 does show improved delivery using SL4 at lower equivalencies of modification (see data at 10eq in Figure 2).

Feedbac #3: For the in vitro study, DSG-PEG was used (page 6). However, PEG-DMG-2000 was used in the in vivo study (page 14). What's the rationale to use these two different PEG lipids?

Response: We selected DSG-PEG for in vitro studies based on some earlier LNP literature that had used this compound in their formulations. However, for in vivo experiments, we opted to switch to DMG-PEG due to its use in LNP formulations for clinical applications (FDA approved Pfizer and Moderna vaccines).

Feedback #4: Please include the LNP characterization with protein cargo in terms of size, zeta potential, and encapsulation efficiency.

Response: A supplemental table (Table S2) containing size, PDI, zeta potential, and encapsulation efficiencies of LNPs encapsulating protein cargos selected for delivery experiments have been included in the revised manuscript.

Q5: The manuscript did not mention Supplementary Fig. 4e-h.

Response: Supplementary Fig. 4e-h have referenced in the manuscript on pages 12 and 13.

Q6: Please be consistent in adding statistical significance to SI figures.

Response: Supplemental figures have been checked to include statistical significance where applicable.

Reviewer: 3

Recommendation: Publish in ACS Central Science without change.

Comments:
This is an excellent study. I think this should be published as is. This is beautifully done!

> Response: We thank Reviewer 3 for their time and effort in reviewing our manuscript and especially for their extremely supportive comments, recognizing that “this is an excellent study” and that our work was “beautifully done!”

oc-2024-00071d.R2

Name: Peer Review Information for "Bioreversible anionic cloaking enables intracellular protein delivery with ionizable lipid nanoparticles"

Second Round of Reviewer Comments

Reviewer: 2

Comments to the Author

In this revised manuscript, the authors provided additional description and information to clarify the experiment procedures and data analysis. All experimental data support the experimental design and conclusions.

Reviewer: 1

Comments to the Author

Thank you for addressing all of my concerns to a high level.

Reviewer: 3

Comments to the Author

Accept as is

Author's Response to Peer Review Comments:

Thank you. We have added a TOC and the corresponding synopsis. Also included a supplementary cover art (also included in the last submission).
